# Cell-Free Demineralized Bone Matrix for Mesenchymal Stem Cells Survival and Colonization

**DOI:** 10.3390/ma12091360

**Published:** 2019-04-26

**Authors:** Monica Mattioli-Belmonte, Francesca Montemurro, Caterina Licini, Iolanda Iezzi, Manuela Dicarlo, Giorgia Cerqueni, Florinda Coro, Giovanni Vozzi

**Affiliations:** 1 Dipartimento di Scienze Cliniche e Molecolari-DISCLIMO, Università Politecnica delle Marche, Via Tronto 10/A, 60126 Ancona, Italy; i.iezzi@pm.univpm.it (I.I.); g.cerqueni@pm.univpm.it (G.C.); 2 Centro di Ricerca “E. Piaggio”, Università di Pisa, Via Diotisalvi 1, 56122 Pisa, Italy; f.montemurro@centropiaggio.unipi.it (F.M.); florindacoro@libero.it (F.C.); g.vozzi@ing.unipi.it (G.V.); 3 Dipartimento di Scienza Applicata e Tecnologia—DISAT, Politecnico di Torino, Corso Duca degli Abruzzi 24, 10129 Turin, Italy; caterina.licini@polito.it; 4 National Institute of Gastroenterology "S. de Bellis", Institute of Research, 70013 Castellana Grotte (BA), Italy; manueladicarlo@alice.it; 5 Dipartimento di Ingegneria dell’Informazione-DII, Università di Pisa, Via Caruso 16, 56122 Pisa, Italy

**Keywords:** decellularized bone matrix, MSCs, gene expression, tissue engineering

## Abstract

Decellularized bone matrix is receiving much attention as biological scaffolds and implantable biomaterials for bone tissue regeneration. Here, we evaluated the efficacy of a cell-free demineralized bone matrix on mesenchymal stem cells (MSCs) survival and differentiation in vitro. The seeding of human umbilical cord-derived MSCs (hUC-SCs) on decellularized bone matrices up to 14 days was exploited, assessing their capability of scaffold colonization and evaluating gene expression of bone markers. Light and Scanning Electron Microscopies were used. The obtained cell-free decalcified structures showed elastic moduli attributable to both topology and biochemical composition. Morphological observation evidenced an almost complete colonization of the scaffolds after 14 days of culture. Moreover, in hUC-SCs cultured on decalcified scaffolds, without the addition of any osteoinductive media, there was an upregulation of Collagen Type I (COL1) and osteonectin (ON) gene expression, especially on day 14. Modifications in the expression of genes engaged in stemness were also detected. In conclusion, the proposed decellularized bone matrix can induce the in vitro hUC-SCs differentiation and has the potential to be tested for in in vivo tissue regeneration.

## 1. Introduction

To develop an efficient and bioactive scaffold with the ability to encourage, guide, and regulate tissue renewal, it is important to consider three main connected factors: 1) the use of a highly biocompatible and bioactive biomaterial; 2) a material easily processable to mime tissue topology; and 3) a scaffold topology fostering the mechanical characteristics of a native tissue in the initial stage of development, as shown by Engler [1].

In recent years, several resorbable synthetic and natural polymers have been explored as biomaterials for bone tissue engineering and regenerative medicine approaches; materials such as poly(lactic acid), polycaprolactone, poly(glycolic acid), polyurethanes, or their blends have been widely used [2,3]. Indeed, they show restricted (e.g., just adhesive groups) or absence of bioactive moieties to improve the biocompatibility and this significantly bounds their regenerative capacity. This weakness can be mainly overcome by either functionalizing the outer surface of scaffolds or just mixing growth factors into the bulk material, so that they can be available with the biomaterial degradation [4]. Biologically-derived proteinaceous materials, such as collagen and fibrin or glycosaminoglycans (e.g., hyaluronic acid), have also been tested [5,6,7,8]. A current method is to arrange different substances to produce a composite structure: Blending bioactive ceramics with polymers improves the mechanical properties of a scaffold, reduces the ceramic brittleness, and increases both scaffolds [9]. Composite scaffolds are consequently expected to be physically and biologically better than single-material based ones. Moreover, the composite features may also be tuned by mixing the different compounds in different ratios since both the composition and relative ratio of constituent materials have an impact on tissue formation. As far as bone is concerned, Hydroxyapatite (HA) has been employed as a key material combined with tricalcium phosphate, poly(lactic-co-glycolic acid) (PLGA), or chitin, to develop different composite scaffolds [10]. Bone scaffolds have been also developed using composite materials based on carbon nanotubes (CNTs) [11,12], which allow to obtain microfabricated scaffolds with good biocompatibility coupled with excellent mechanical and electrical properties [13,14]. Moreover, synthetic polymers, such as PLGA, were combined with bioglasses, developing scaffolds with an elastic modulus in the range of natural bone tissue, and endowed with a good cell interaction also in term of stem cell osteoblastic differentiation [15].

Tissue decellularization was first reported in 1973 [16] as a method to preserve tissue planned as a protective barrier for burn patients. The biochemical composition and 3D organization of the Extracellular Matrix (ECM) is characteristic for each tissue type and it entails the functional molecules secreted by tissue resident cells as well as the tissue structure [17]. Since ECM is capable to dynamically and reciprocally influence cell behavior [18], over the past several decades the mimicking of the composition and structure of the ECM has directed the coherent design of biomaterials in attempts to properly influence cell performances [19,20]. The increased interest to use ECM derived from the decellularization of tissues or organs as biomaterial allowed to develop functional tissues such as skin, bladder, heart valve and small intestinal submucosa [21]. 

As far as bone tissue regeneration is concerned, the “gold standard” is represented by bone graft that, encompassing low levels of bone morphogenetic proteins (BMPs), is osteoconductive [22]. Bone decellularization and demineralization result in a spongy deformable scaffold, maintaining osteoconductive properties, that can be used as a bone void filler and as a matrix for cells. Therefore, the use of decellularized bone matrix as scaffolds could furnish tissue-specific ECM cues that influences the behavior of resident and/or seeded cells. Indeed, the actual impact of the ECM mechanics of a cell-free and demineralized scaffold—regardless the microstructure - on the osteogenic differentiation of endogenous osteoprogenitor cells, which arrange bone regeneration and repair, is still to be completely elucidated [23].

In a decellularization process there are still concerns on the need to remove each cell component to avoid disease transmission, inflammation and/or immune responses towards the scaffold, and decrease the risk of rejection after implantation. Moreover, it is mandatory to optimize reagents and time for decellularization, to remove only cell components without affecting bio-cues promoting bone cell activities and functions. 

Based on these considerations, we have optimized the protocol of decellularization and demineralization of bovine cancellous bone proposed by Eagle and Collagen Type I (COL1). [24] to produce scaffolds not only endowed by chemical bone matrix cues [25] but also with the topological features of a native bone tissue. These scaffolds were then seeded human mesenchymal stem cells (MSCs) derived from umbilical cord (hUC-SCs) to evaluate if they provide a suitable environment for cell adhesion and maintenance.

## 2. Results 

### 2.1. Bone Demineralization and Mechanical Tests

**Figure 1:**
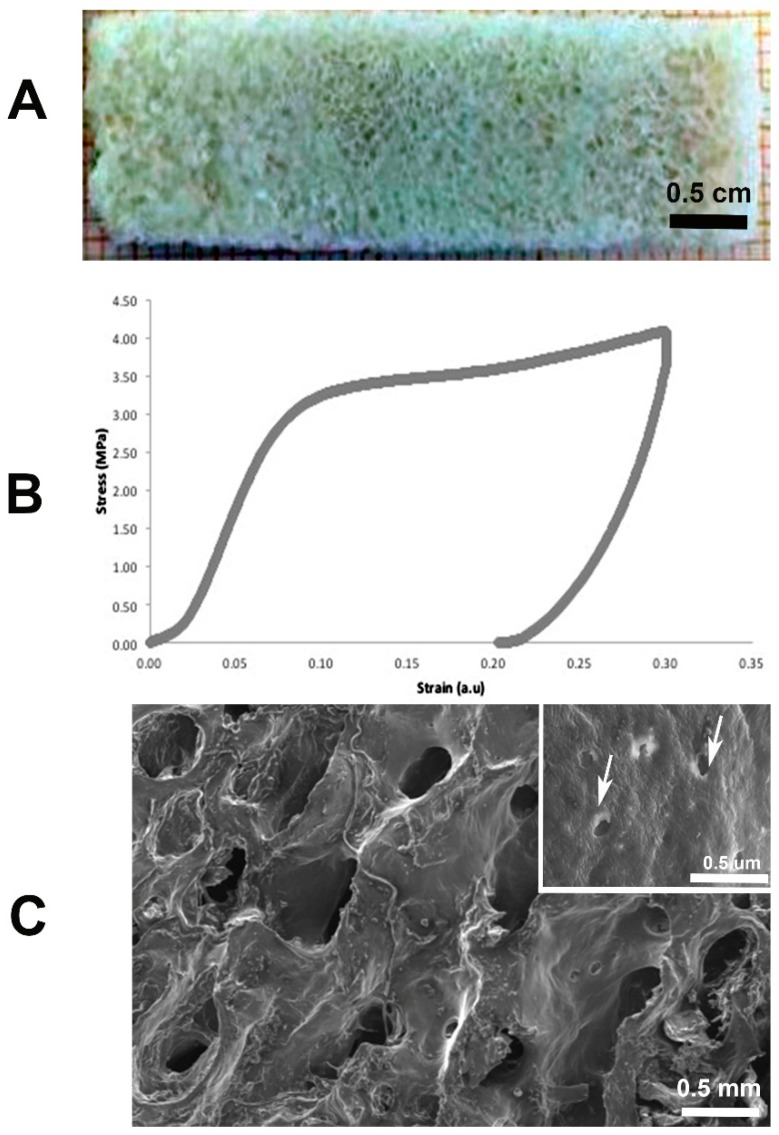
Representative: (**A**) Demineralized scaffold; (**B**) stress-strain curve of demineralized bone; (**C**) SEM observation with bony trabecular network and empty osteocytic lacunae (arrows).

Decalcification ended after the second day of immersion in decalcifying solution, as shown by decalcification test where no precipitate was found. Figure 1 shows the morphological appearance, as well as an example of a characteristic compressive stress-strain curve, of the obtained demineralized sample. As it is shown in the graph, it is possible to determine two elastic moduli: the one for low strains is principally due to the topology of the demineralized bone scaffold, whilst the elastic modulus for high strain is relative to the biochemical composition of the scaffolds. The obtained elastic moduli values were 2.91 ± 2.3 MPa and 23.25 ± 7.54 MPa, respectively. Data obtained were in line with literature [26]. Scanning electron microscopy (SEM Philips XL 20, FEI Italia SRL, Milan, Italy) observation and Hematoxylin-Eosin staining demonstrated a bony trabecular network with the absence of cells.

### 2.2. Isolation and Characterization of Human Umbilical Cord Stem Cells (hUC-SCs)

**Figure 2:**
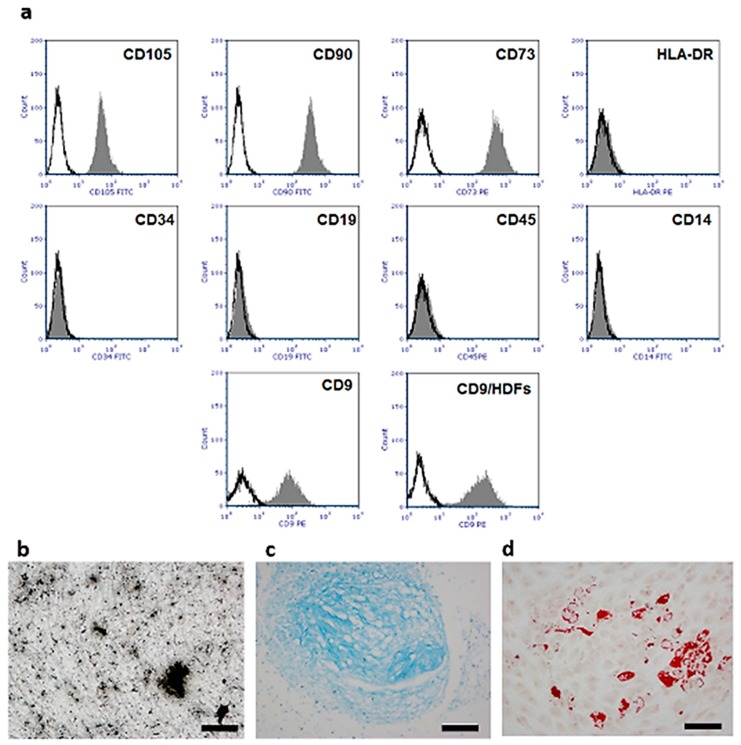
hUC-SC flow cytometric phenotypic characterization and in vitro differentiation assays. (**a**) Characteristic single-variable histograms display negative control (black line) and the expression of cell surface antigens (grey) on hUC-SCs and Human Dermal Fibroblasts (HDFs); (**b**) osteogenic differentiation of hUC-SCs after 21 days of induction (Von Kossa staining); (**c**) chondrogenic differentiation of hUC-SCs after 14 days of induction (Alcian Blue staining); (**d**) adipogenic differentiation of hUC-SCs after 14 days of induction (Oil Red staining). Image magnification 20×, scale bars 50 μm.

Evaluation of hUC-SC surface markers were made by flow cytometry at the 3rd cell passage of subculture. Cells expressed CD73, CD90, and CD105 antigens and were negative for CD34, CD45, HLA-DR and CD14. According to the minimal criteria recommended by the International Society for Cellular Therapy [27], our cells could be considered to be MSCs. Besides, our cell population displayed a lower expression level of CD9 with respect to Human Dermal Fibroblasts (HDFs), with a Mean Fluorescence Intensity (MFI) of 94.18 in hUC-SCs and 167.76 in HDFs. This data confirms that the analyzed cell populations were homogenous with no fibroblast contamination [28] (Figure 2a). In vitro differentiation assays demonstrated the ability of hUC-SCs to differentiate, in appropriate culture conditions, into chondrocytes, osteoblasts, and adipocytes (Figure 2b–d), strengthening their belonging to the MSCs family.

### 2.3. Scaffold Colonization 

SEM observation at 7 days of culture evidenced the colonization of cell-free demineralized structures by hUC-SCs, with randomly disperse cells on the surface of the scaffolds, as well as within the macropores. Most cells have an elongated, fibroblast-like appearance. 

**Figure 3:**
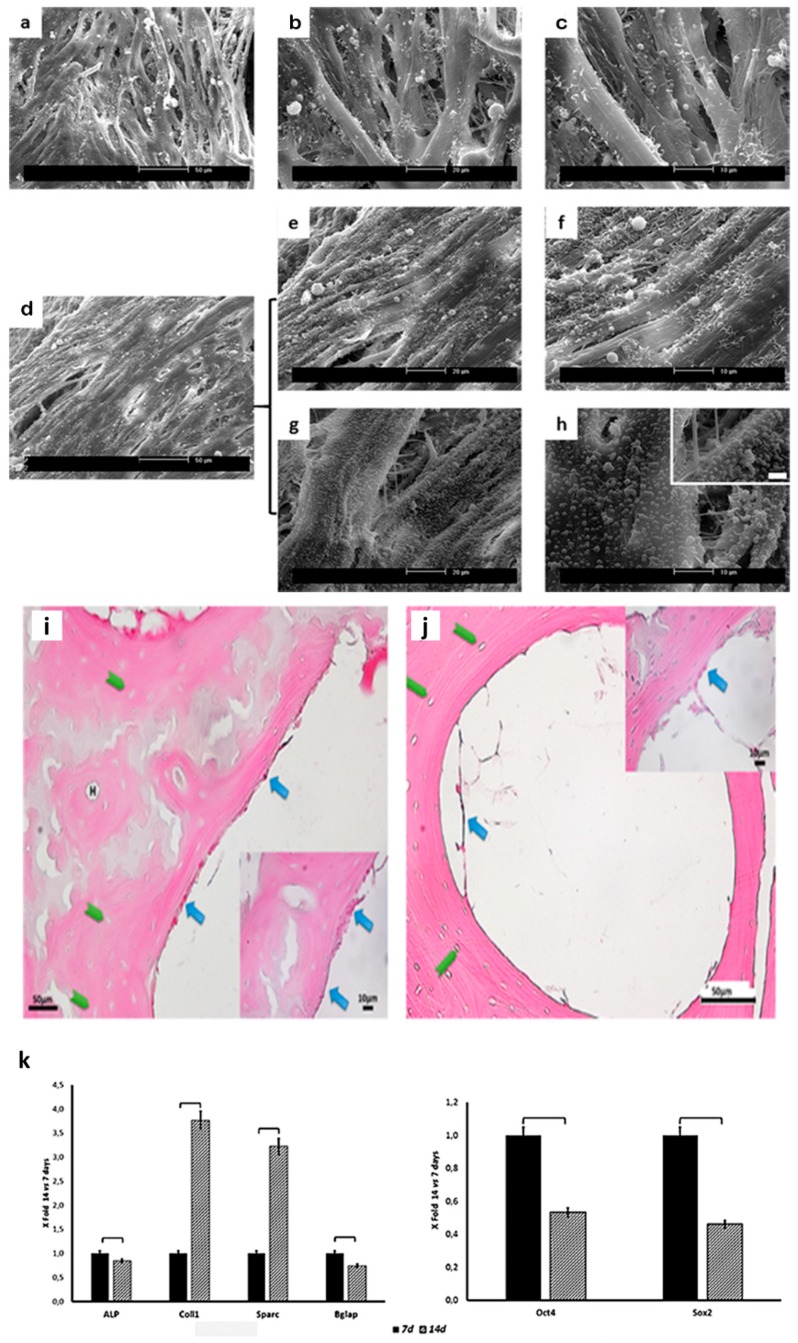
SEM micrographs of scaffolds cultured with hUC-SCs for 7 (**a–c**) and 14 (**d**–**h**) days at different magnifications. Cells were able to adhere and colonize the scaffolds. Note the presence of membrane blebs after 14 days of culture suggestive of active metabolizers. Light microscopy (LM) micrographs of cells on demineralized scaffold at 7 (**i**) and 14 (**j**) days of culture: H = Haversian canal; green arrows indicate osteocytic lacunae; blue arrows indicate hUC-SCs; (**k**) histograms depict changes in Alkaline Phosphatase (ALP), Collagen Type 1 (COL1), ostenectin (ON; also known as secreted protein acidic and rich in cysteine (SPARC)), osteocalcin (OC; also known as bone gamma-carboxyglutamic acid-containing protein (BGLAP), sex-determining region Y (SRY)-Box2 (Sox2), and octamer-binding transcription factor 4 (Oct4) mRNA expression in hUC-SCs. Data are expressed as fold-change (2^−ΔΔCt^) of the expression at 14 days culture over 7 days one: square brackets indicate significant differences (*p* < 0.05).

At 14 days, scaffolds were covered with a multilayered canopy of cells growing inwardly. Cellular boundaries were indiscernible due to the flattened morphology and intimate contact between neighboring cells. Blebbing on cell surface was also evident (Figure 3a–h). 

Light microscopy (LM, Nikon Eclipse 600, Nikon Instruments Spa, Florence, Italy) images were consistent with SEM observation at both time points analyzed with cells adhering the surface of the scaffolds (Figure 3i,j).

### 2.4. Gene Expression

Data on mRNA relative expression in the different subculture passages are summarized in Table 1.

A significant increase in COL and osteonectin (ON), i.e., secreted protein acidic and rich in cysteine (SPARC)), mRNA expression was detected after 14 days of culture in comparison with cells cultured for only 7 days. On the contrary, a significant decrease was noticed in the expression of Alkaline Phosphatase (ALP) and of the latest marker of osteoblastic differentiation (i.e., osteocalcin (OC; also known as bone gamma-carboxyglutamic acid-containing protein (BGLAP) between the two time-point analyzed. As far as gene involved in stemness is concerned (i.e., octamer-binding transcription factor 4 (Oct4) and sex-determining region Y (SRY)-Box2 (Sox2), a significant decrease in both genes’ mRNA expression was detected after 14 days of culture. It must be stressed that gene expression was significantly lower of that detected in cells cultured in tissue culture plates (TCPs) (data not shown). 

These changes were also analyzed using the ΔΔCt method [29], comparing mRNA expression of hUC-SCs cultured for 14 days with that of cells after 7 days of culture (Figure 3k). A 3-fold increase in COL1 and ON mRNA expression was observed in cells cultured for 14 days on the demineralized scaffolds in comparison with those cultured for 7 days. Concomitantly, there was a reduction in the expression of gene involved in the maintenance of self-renewal profile (Figure 3k). 

## 3. Discussion

Biological scaffolds composed of ECM and derived from different mammalian tissue decellularization are broadly utilized in clinical applications involving the healing and regeneration of tissues and organs (e.g., musculoskeletal tissues, gastrointestinal or lower urinary tract) [30]. The goal of tissue decellularization is the exhaustive elimination of cells and debris while maintaining, as much as possible, the composition and 3D ultrastructure of the native ECM. The initial studies on tissue decellularization removed cellular material and retained the structural and functional proteins of the ECM, such as glycosaminoglycans (GAGs), proteoglycans, and growth factors [31]. ECM materials harvested by such methods and appropriately processed preserved the biochemical complexity, nanostructure, and bioinductive properties of the natural matrix, being able to encourage the in vivo creation of site-specific, functional tissue [32]. Several ECM-derived materials are FDA-approved, can be conserved and used ‘off the shelf,’ have been extensively characterized in both the 2D sheet and powder forms, and have been implanted in millions of patients to date [32,33,34]. 

The ideal methods of decellularization depends on tissue-specific factors, such as cell number, matrix density, and geometric thoughts, including tissue thickness and shape. The whole removal of all cell fragments is not possible and, predictably, decellularization procedures can cause some alteration in matrix architecture, orientation, and surface ligand sites. Therefore, micro-architectural features, which include ECM fiber orientation, connectivity, intersection spatial density, and diameter (i.e. network topology), are not always well preserved during these processes. As far as bone is concerned, this decellularization needs to be optimized to avoid the removals of biocues supporting bone cell activities and coupling mechanisms between osteoclasts and osteoblasts. The produced structures, able to encourage a tissue remodeling, must also favor the recruitment and differentiation of stem/progenitor cells [24,35]. 

In this respect, the proposed decellularized scaffolds evidenced a good growth and colonization by hUC-SCs. These cells are attractive for several tissue engineering applications, as they are derived from an ethically undisputed source and may be non-invasively harvested at low cost [36,37,38,39]. Our observation is in line with data of Xu et al. [40], who showed a good proliferation for hUC-SCs cultured on medium-stiffness matrices. In their work, the authors also evidenced that on the medium-stiffness matrices, which were comparable to pre-mineralized bone, hUC-SCs presented a spindle-shaped cells similar in morphology to myoblasts rather than osteoblast as described in Engler’s work [41]. Even if cell shape is not necessarily suggestive of stem cell differentiation, our scaffolds’ cells showed a spindle shaped morphology with evident blebs, which were suggestive of an osteoblastic induction.

Gene expression results showed a reduction of mRNAs involved in self-renewal, indicating the capability of the proposed scaffolds to start cell differentiation also in the absence of any other microenvironmental signal. Indeed, cells showed an increase only of COL1 and ON, whilst genes involved in mineralization seemed to be downregulated. This feature could be at least in part attributable to the lack of appropriate differentiation cues, in addition to material stiffness, that could induce the expression of ALP and OC. Comparable data were obtained by Witkowska-Zimny et al.’s research, who did not show changes in the expression of ALP during UC-SCs osteogenic differentiation on matrix scaffold [42]. In addition, differences could also exist between UC-SCs and other sources of MSCs, such as periosteal derived stem cells, in terms of osteogenic potential [23,43].

According to the obtained results, the proposed scaffolds obtained by bovine cancellous bone could be suitable tools for the in vitro testing of cell behavior due to the inductive effects of ECM- derived molecules in term of adhesion, growth, and differentiation. 

Our results also emphasize the importance of topography as a determinant of cellular behavior within a porous substrate. More investigations are still required to elucidate the exact effects of this scaffold and its interactions with MSCs.

## 4. Materials and Methods

### 4.1. Bone Demineralization and Mechanical Tests

We obtained three-dimensional (3D) bone spongy scaffolds from bovine cancellous bone, obtained from the femur of 18-month-old bovines (2 males and 2 females) [44]. Slices of 40 mm × 15 mm and of 2–3 mm of thickness were first washed with sodium hypochlorite and then demineralized using 2% (w/v) HCl 0.5N solution in water (Sigma, Milan, Italy) about 50 ml for gram of bone [24]. Bath solution was changed every day and decalcification was evaluated mixing one part of bath solution with two parts of a solution made of equal part of 5% (w/v) NH_3_ and 5% (w/v) Ammonium Oxalate [45]. Decalcification was stopped after 24 h if they were not precipitate. Samples were then washed in ultrapure water and allowed to dry before performing mechanical tests. 

Young’s modulus of 3D bone scaffolds (six samples) was evaluated using uniaxial testing machine Zwick/Roell mod. Z005, equipped with a 100 N cell load (Zwick Roell Italia S.r.l., Genova, Italy). Compression tests were performed with the following parameters: 0.1 N of preload, strain rate equal to 1% of sample thickness, and 30% of maximum deformation. 

Data were analyzed with Excel, and Young’s modulus was obtained from linear regression of initial portion of stress-strain curve. Samples were also fixed in 10% neutral buffered formaldehyde (Diapath S.P.A., Martinengo, Italy) for at least 2 days and processed according to standard histology (see below).

### 4.2. Isolation and Culture of Human Umbilical Cord Mesenchymal Stem Cells (hUC-SCs)

hUC-SCs were isolated from Wharton’s jelly of 5 healthy full-term pregnancies after women informed consent obtainment. Samples were collected in agreement with the guidelines of the National Bioethics Committee and handled following a procedure approved by the University of Bologna. Briefly, umbilical cords were finely minced and subjected to enzymatic digestion with 0,1% (w/v) collagenase (Gibco, Thermo Fisher, Monza, Italy) in phosphate buffer saline (PBS) for 3 h at 37 °C in a humidified incubator. Digestion products were filtered by a sterile cell strainer (70 μm in diameter) to remove ECM debris. The filtrate was then washed several times with PBS supplemented with 10% (v/v) fetal bovine serum (FBS) (Gibco, Thermo Fisher) and, at last, cultured in Dulbecco’s modified eagle medium/nutrient mixture F-12 (DMEM/F12) (Life Technologies, Milan, Italy) containing 10% FBS and 1% penicillin–streptomycin (100 U/mL) in cell culture flasks to allow the adhesion of primary hU-SCs. Culture medium was completely replaced twice a week. Cells were detached at 80–90% confluency with 0.25% trypsin in 1mM ethylenediaminetetraacetic acid (EDTA) (Gibco, Thermo Fisher) and split 1:2. 

The staminal profile of 3rd passaged hUCs was assessed by flow cytometry considering the minimal criteria for the identification of human MSCs [27]. To this aim 2.5 × 10^6^ cells were aliquoted in round-bottom polystyrene tubes, washed with PBS and then stained for 45 min at 4 °C with mouse anti-human FITC (Fluorescein isothiocyanate) or PE (Phycoerythrin)-conjugated monoclonal antibodies against the following surface antigens: HLA-DR, CD34, CD105, CD14, CD19, and CD45 (Diaclone, Besancon, France); CD73 and CD90 (StemCell Technologies, Inc. Vancouver, BC, Canada) and CD9 (Thermo Fisher). HDFs were used to compare CD9 expression levels. Control for FITC- or PE-coupled antibodies was an isotypic mouse IgG1. Flow cytometric analysis was performed using FACSCalibur flow cytometry system (Becton Dickinson, CA, USA) and FCS Express 6 Plus Software (De Novo Software, Los Angeles, CA, USA).

### 4.3. hUC-SC In Vitro Differentiation

At the 3rd passage, the ability of hU-SCs to differentiate into mesenchymal lineages was evaluated. For osteogenic differentiation, hU-SCs were plated at the density of 5 × 10^4^ cells with STEMPRO^®^ Osteogenesis Kit in chamber-slides (Lab-Tek II Chamber Slide, Nalgene Nunc International, Thermo Fisher). After 21 days, Von Kossa staining was performed to assess the appearance of calcium deposits. Briefly, cells were fixed in 4% paraformaldehyde (PFA) for 15 min at room temperature (RT) and incubated with a 1% silver nitrate solution under UV light for 20 min at RT. Unreacted silver was removed with 5% sodium thiosulfate solution for 5 min. To assess the differentiation into adipocytes, 4 × 10^4^ hU-SCs were seeded in chamber-slides with STEMPRO^®^ Adipogenesis Kit and after 15 days Oil Red staining was performed to visualize lipid droplets. Cells fixed in 4% PFA were washed two times with PBS and one time with isopropanol 60%. After incubation with Oil Red O solution 0.5% (w/v) in absolute isopropanol diluted 3:2 with dH_2_O for 30 min at RT, cells were washed in dH_2_O and counterstained with Mayer’s hematoxylin (BioOptica, Milan, Italy). For chondrogenesis, pellet culture system was used. In brief, 1 × 10^6^ cells in 1mL of STEMPRO^®^ Chondrogenesis Kit were centrifuged at 1200 rpm for 5 min. Pellets were cultured for 14 days and then fixed in 4% PFA, paraffin embedded, and sectioned. Sections were incubated with a solution of Alcian Blue pH 1 (Bio-Optica, Milan, Italy) for 20 min at RT and then washed with dH2O. Previous kits were all purchased from Gibco (Thermo Fisher). All the reactions were examined with a light microscope (Nikon Eclipse 600) equipped with a Nikon DSVi1 digital camera (Nikon Instruments) and NIS Elements BR 3.22 imaging software (Nikon Instruments).

### 4.4. Scaffold Seeding and Culture

Before seeding, demineralized 3D scaffolds, placed in Corning^®^ ultra-low attachment multiwell plates were sterilized at each side under UV for 30 min and then incubated in 70% ethanol (Sigma-Aldrich, Milan, Italy) for 1 h. Scaffolds were then conditioned by rinsing in complete DMEM/F12 (supplemented as previously described) overnight in a humified incubator (37 °C at 5% CO_2_). After media removing, scaffolds were considered ready for cell seeding. hUC-SCs were detached from culture flasks using 0.25% trypsin in 1mM EDTA and seeded on samples at a density of 2x10^5^ cells/sample in a volume of 50 μL. After 1 h of incubation at 37 °C, scaffolds were fixed with CellCrown™ (Scaffdex, Tampere, Finland) to prevent sample floating, and 1 ml of complete DMEM/F12 was added in each well. hUC-SCs were further cultured on the demineralized 3D scaffolds for 7 and 14 days. Media were changed twice a week. hUC-SCs cultured at a density of 2 × 10^5^ cells/well on TCPs for 7 and 14 days were used as controls for real-time quantitative reverse transcription (qRT)-PCR assays.

### 4.5. Scanning Electron Microscopy (SEM)

For SEM observation, cell-seeded demineralized 3D scaffolds were fixed in 2.5% glutaraldehyde in 0.1 M cacodylate buffer, post-fixed in 1% osmium tetroxide, dehydrated in increasing ethanol concentrations (25, 50, 70, 80, and 100%) and hexamethyldisilazane (HDMS)-dried, mounted on aluminum stubs, gold-sputtered by the Edwards Sputter Coater B150S equipment and observed with a Philips XL 20 SEM (FEI Italia SRL, Milan, Italy) microscope. All reagents were purchased from Sigma-Aldrich.

### 4.6. Light Microscopy (LM)

For LM, cultured specimens (7 and 14 days) were fixed in 10% buffered formaldehyde (Diapath S.P.A) embedded in paraffin (AppliChem GmbH, Darmstadt, Germany), sectioned to a thickness of 5–6 µm and stained with Hematoxylin-Eosin (Bio-Optica S.P.A., Milano, Italy) for routine histological examination. The sections were dewaxed in xylene (Carlo Erba Reagents, Val-de-Reuil, France) and rehydrated through a graded series of ethanol (Carlo Erba Reagents).

### 4.7. Real-Time Quantitative Reverse Transcription PCR (qRT-PCR)

Total RNA was retrieved from cells cultured for 7 and 14 days on decellularized bone scaffolds and TCPs with TRIzol^®^ Reagent (Invitrogen, Milan, Italy), according to the manufacturer’s instructions. RNA quantification and the evaluation of its quality were made by spectrophotometric analysis (bioPhotometer plus, Eppendorf GmbH, Hamburg, Germany): 2.5 µg of total RNA was reverse transcribed in a 20 µL reaction volume using the SuperScript IV VILO Master Mix (Thermo Fisher). Neo-synthesized cDNA was kept at −20 °C. 

Mastercycler Realplex2 thermocycler (Eppendorf GmbH) was used for real-time assays with SsoFast™ EvaGreen^®^ Supermix 1× in a final volume of 10 µL. All PCR reactions included 1 µL of cDNA (equivalent to 50 ng of total RNA template). Primer sequences were designed by Primer 3 (v. 0.4.0, ThermoFisher^®^Primers) software and each primer was used at a 200nM final concentration. To circumvent any substantial homology to pseudo-genes or other unexpected targets, primer specificity was tested by BLAST Assembled RefSeq Genomes. Table 2 depicts oligonucleotide sequences for target and reference genes. mRNA of both reference genes and each gene of interest were measured under matching conditions and at the same time in each assay. Primers exhibited equal amplification efficiency. Specificity of the PCR reactions was also determined by melt curve analysis: For each amplicon, the detected melting temperature was the expected one.

Threshold Cycle (Ct) values for reference genes were utilized to normalize cell mRNA data. Each assay was made in triplicate. Normalization involved the ratio of mRNA concentrations for specific genes of interest (as mentioned above) to that corresponding to Ct medium values for glyceraldehyde-3-phosphate dehydrogenase (GAPDH) and beta glucuronidase (GUSB) [46]. Data were expressed as gene relative expression (2^−ΔCt^). To point out the effect of scaffolds on hUC-SCs, ΔΔCt method for Fold-Change evaluation was used comparing values obtained in cells cultured for 14 days with those seeded for only 7 days [29]. The qPCR efficiency in all our experiments was more than 90%. The difference between the actual and theoretical (100%) efficiencies would result in an underestimation of the mRNA concentration of all the analyzed samples.

### 4.8. Statistical Analysis

SAS statistical package (Statistical Analysis System Institute) was used. All experiments were carried out in triplicate and results are expressed as mean ± Standard Deviation (SD). ANOVA and Bonferroni tests were used to analyze the differences. Statistical significance was set at *p* < 0.05.

## Figures and Tables

**Table 1:** mRNA relative expression.

Genes	7 Days		14 Days		
	Mean	DS	Mean	DS	*p*
ALP	2.93 × 10^−4^	1.47 × 10^−5^	2.46 × 10^−4^	1.23 × 10^−5^	0.039
COL1	1.33 × 10^−1^	6.64 × 10^−3^	4.99 × 10^−1^	2.50 × 10^−2^	0.0096
SPARC	1.52 × 10^−1^	7.59 × 10^−3^	4.89 × 10^−1^	2.44 × 10^−2^	0.013
BGLAP	4.96 × 10^−6^	2.48 × 10^−7^	3.64 × 10^−6^	1.82 × 10^−7^	0.047
Oct4	9.79 × 10^−4^	4.89 × 10^−5^	5.21 × 10^−4^	2.60 × 10^−5^	0.034
Sox2	3.93 × 10^−5^	1.97 × 10^−6^	1.77 × 10^−5^	8.85 × 10^−7^	0.042

**Table 2:** Analyzed gene description.

Genes	Detected Transcript	Primer Forward (5′→3′)	Primer Reverse (3′→5′)	Amplicon Length (bp)
**ALP**	NM_007431	GGCCAGCTACACCACAACA	CTGAGCGTTGGTGTTATATGTCTT	96
**COL1**	NM_000088.3	CCAACCCTTCCACCTTTGGAAGT	CCGGAGGTCCACAAAGCTGAA	132
**SPARC**	NM_003118.3	CCTGAGGCTGTAACTGAGAGAAAG	GTGGGAGGGGAAACAAGAAGATAA	142
**BGLAP**	NM_199173	GACTGTGACGAGTTGGCTGA	GCCCACAGATTCCTCTTCTG	119
**Sox2**	NM_003106.3	ACACCAATCCCATCCACACT	GCAAACTTCCTGCAAAGCTC	198
**Oct4**	NM_203289.4	AGCGAACCAGTATCGAGAAC	GCCTCAAAATCCTCTCGTTG	199
GUSB *	NM_000181.2	AAACGATTGCAGGGTTTCAC	TCTCGTCGGTGACTGTTCA	81
GAPDH *	NM_002046.3	AGCCACATCGCTCAGACAC	GCCCAATACGACCAAATCC	200

* Reference genes: GUSB = beta glucuronidase; GAPDH = glyceraldehyde-3-phosphate dehydrogenase.
